# VX680 suppresses the growth of HepG2 cells and enhances the chemosensitivity to cisplatin

**DOI:** 10.3892/ol.2013.1648

**Published:** 2013-10-29

**Authors:** RUCHENG YAO, JUN ZHENG, WEIHONG ZHENG, YUAN GONG, WEI LIU, RONGCHUN XING

**Affiliations:** 1 Department of General Surgery, The First College of Clinical Medical Sciences, Three Gorges University, Yichang, Hubei 443003, P.R. China; 2 Department of Pharmacology, Medical Science College, Three Gorges University, Yichang, Hubei 443003, P.R. China; 3 Department of Respiratory Medicine, The First College of Clinical Medical Sciences, Three Gorges University, Yichang, Hubei 443003, P.R. China

**Keywords:** Aurora A, chemosensitivity, cisplatin, HepG2 cells, VX680

## Abstract

VX680 is an Aurora A inhibitor. It has been reported to inhibit the growth of the HepG2 cell line in several studies. However, whether it enhances chemosensitivity to cisplatin remains unclear. In this study, the synergistic effect of VX680 and cisplatin on the proliferation of HepG2 cells was determined by MTT assay. The changes in cell apoptosis were detected by flow cytometry. Aurora A, Bcl-2 and p53 protein levels were analyzed by western blotting. This study demonstrated that VX680, cisplatin and a combination of the two inhibit the growth of HepG2 cells in a dose- and time-dependent manner. A synergistic effect was observed with the combined therapy. Moreover, the inhibitory effect of VX680 was positively correlated with the expression of Aurora A. The rate of apoptosis in the combined group was significantly higher compared with that of the VX680 and cisplatin groups. In addition, VX680 and cisplatin increased the expression of the p53 protein. Cisplatin reduced the expression of Bcl-2 protein, while VX680 did not. In the combined group, the expression of Bcl-2 and p53 changed significantly compared with the single drug group and control group. This study suggests that Aurora A may represent a valid target in hepatocellular carcinoma. We also demonstrated that the Aurora A inhibitor VX680 has a synergistic effect with cisplatin.

## Introduction

Hepatocellular carcinoma is one of the most common malignancies in humans that severely threatens people’s health. Surgical therapy is the most effective method for patients who suffer from non-advanced hepatic carcinoma (1). However, the majority of patients with hepatocellular carcinoma have poor prognosis and succumb within several months of diagnosis. Traditional chemotherapy is often used in patients with unresectable hepatocellular carcinoma. However, common problems include the severe toxicity to normal tissue and the high resistance to the majority of chemotherapeutic drugs. Therefore, a drug with low toxicity that is relatively selective for cancer cells and has a synergistic effect with chemotherapeutic drugs is extremely important. It is the key to increasing the survival rate of liver cancer patients, particularly for advanced patients.

The Aurora kinase family consists of serine/threonine kinases (2). They are critical in regulating the majority of mitotic processes and are frequently highly expressed in human cancers. Increased cellular levels of these kinases may be related to genetic instability and are evident in various cancer types, including breast, ovarian, colon and pancreatic cancer. In mammalian cells, according to their location, Aurora kinases are divided into three types: Aurora A, Aurora B and Aurora C.

A number of studies have demonstrated that Aurora A and Aurora B are overexpressed in lung cancer (3), colorectal cancer (4), prostate cancer (5), renal carcinoma (6), hepatocellular carcinoma (7), ovarian cancer (8) and bladder cancer (9). Enhancing their expression causes cell mitotic errors, cell malignant transformation and genome instability. By contrast, suppressing their expression inhibits cell proliferation and promotes cell apoptosis (10). Therefore, the Aurora kinase family members have become potentially valuable antitumor therapeutic targets.

A number of Aurora kinase inhibitors have been discovered (11,12), including VX680, ENMD-2076, ZM447439 and MLN8237. VX-680 has been shown to disrupt mitosis and induce apoptosis in a wide variety of tumor cell lines (13). VX-680 was also the foremost Aurora kinase inhibitor to be studied in clinical trials (14). The clinical studies of Aurora kinase inhibitors have already reached phase II trials; however, their potential application in the treatment of hepatocellular carcinoma (HCC) remains to be investigated.

In the present study, we aimed to determine whether VX680 is able to effectively reduce the toxicity of cisplatin chemotherapy and effectively inhibit the growth of hepatoma cells. Accordingly, we first used VX680, cisplatin and a combination of the two to explore their effects on HepG2 cells. Then, we investigated the effect and mechanism of VX680 on the growth inhibition of HepG2 cells, and the synergistic effect with cisplatin.

## Materials and methods

### Cell and reagents

The HepG2 cell line was kindly provided by the Medical College of Three Gorges University (Yichang, China). The cells were cultured in RPMI-1640 (HyClone, Logan, UT, USA) supplemented with 10% fetal bovine serum and 100 U/ml penicillin/streptomycin at 37°C in a humidified atmosphere containing 5% CO_2_. After cell growth reached 70–80% confluency in the bottom of the culture bottle, logarithmic phase cells were used for the experiment. VX680 was purchased from Selleck Chemicals (Houston, TX, USA), and was dissolved in dimethyl sulfoxide (Sigma-Aldrich, St. Louis, MO, USA), stored at −80°C and diluted in fresh medium immediately before use. Cisplatin was purchased from Qilu Pharmaceutical Co., Ltd. (Shandong, China).

### 3-(4,5-Dimethylthiazol-2-yl)-2,5-diphenyltetrazolium bromide (MTT) assay for cell growth inhibition

Logarithmic phase cells were cultured in 96-well plates and treated with varying doses of VX680 (3.125–50 μmol/l) and cisplatin (0.125–2 μg/ml) for 24–72 h at 37°C in a humidified atmosphere containing 5% CO_2_. Following incubation with 20 μl MTT (5 mg/ml) for 4 h, 150 μl DMSO was added to each well. Subsequently, the 96-well plates were agitated for 15 min at micro-oscillator oscillation. The optical density (OD) value at 490 nm was measured by automatic enzyme-linked immunosorbent assay readers. The inhibition rate was calculated using the following equation: (1 − average OD value of experimental group/average OD value of control group) ×100. Whether the two drugs had synergistic or antagonistic effects was determined according to the following formula (15): Q = E(A + B)/[(EA + EB) − (EA × EB)], where a Q-value of 0.85–1.15 indicates the sum of the effects and a Q-value >1.15 indicates a synergistic effect. By contrast, a Q-value <0.85 indicates the antagonistic effect of the combined drugs. EA represents the inhibition rate for drug A, EB represents the inhibition rate for drug B and E(A + B) represents the inhibition rate for the combined therapy.

### Apoptosis detected by flow cytometry

Cells (1×10^6^/ml) were cultured in six-well plates for 24 h and then treated with VX680 (3.125 μmol/l), cisplatin (0.5 μg/ml) or VX680 (3.125 μmol/l) and cisplatin (0.5 μg/ml) for 72 h. Cells with no drugs added were used as the control. Apoptosis was detected according to the Annexin V-FITC Apoptosis Detection kit (BD Transduction Laboratories, Lexington, KY, USA). Cells (1×10^5^/ml) were centrifuged at 1,200 × g for 5 min, then the supernatant was removed. Later, the cells were treated with 195 μl Annexin V-FITC conjugation liquid. After adding a further 5 μl Annexin V-FITC, the cells were incubated at room temperature for 15 min. The above steps were repeated two times. After staining with Annexin V-FITC away from light, 10 μl propidium iodide was added and cells were analyzed using a BD Accuri C6 flow cytometer (BD Biosciences, Ann Arbor, MI, USA). Data were processed and analyzed using the Accuri CFlow Plus software, version 1.0.227.4 (BD Biosciences).

### Western blot analysis

HepG2 cells (5×10^6^/ml) were cultured with VX680 (3.125 μmol/l), cisplatin (0.5 μg/ml) and VX680 (3.125 μmol/l) plus cisplatin (0.5 μg/ml) for 72 h. Following this, the cells were washed with cold phosphate-buffered saline and lysed with radio-immunoprecipitation assay buffer (Beyotime, Shanghai, China). The protein concentration was measured by a bicinchoninic acid protein assay kit (Pierce, Rockford, IL, USA). Fifty micrograms of total protein were denatured by boiling for 5 min, then separated using 10% sodium dodecyl sulfate-polyacrylamide gel electrophoresis and transferred onto a nitrocellulose membrane (Millipore Corp., Boston, MA, USA). The blots, with 5% non-fat milk powder and 1 ml/l Tween-20/Tris-buffered salt solution (TTBS), were blocked for 2 h, followed by incubation with the primary antibodies (mouse monoclonal; 1:500 dilution) for Aurora A (Abcam, Cambridge, MA, USA), Bcl-2, wt p53 (Santa Cruz Biotechnology, Inc., Santa Cruz, CA, USA) and β-actin (Wuhan Boster Biological Technology, Ltd., Wuhan, China) for 2 h at room temperature. After extensive washing with TTBS, the blots were incubated with a monoclonal secondary mouse IgG antibody (1:5,000; Wuhan Boster Biological Technology, Ltd.) for 1 h and washed with TTBS. Protein bands were analyzed by SmartView gel imaging system (Shanghai Furi Technology Co., Ltd., Shanghai, China).

### Statistical analysis

Data were analyzed by SPSS version 13.0 software (SPSS Inc., Chicago, IL, USA) and were expressed as the mean ± SD. A single-factor analysis of variance was used to compare the differences between groups. For all analyses, P<0.05 was considered to indicate a statistically significant difference.

## Results

### Effect of VX680 and cisplatin on the proliferation of HepG2 cells

**Figure 1:**
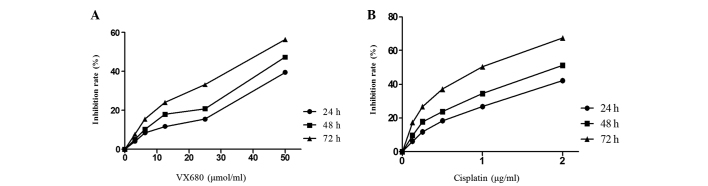
Hepatocellular carcinoma (HepG2) cells were treated with varying concentrations of VX680, cisplatin and a combination of the two. The growth inhibition rate of HepG2 cells was assessed by 3-(4,5-dimethylthiazol-2-yl)-2,5-diphenyltetrazolium bromide assay. Synergy between these groups was assessed by Q-value. (A and B) Different concentrations of drugs (VX680, 3.125–50 μmol/l; cisplatin, 0.125–2 μg/ml) and treatment times had various effects on the cell growth inhibition rate.

Following culture with VX680 or cisplatin, HepG2 cellular proliferation was monitored by MTT assay daily for 24, 48 and 72 h. Cell proliferation was significantly suppressed by VX680 and cisplatin in a time- and dose-independent manner (Fig. 1).

In order to determine whether VX680 synergistically enhances the effect of cisplatin, HepG2 cells were cultured with 3.125 μmol/l VX680 (10% cytotoxicity) and cisplatin (0.125–2 μg/ml) for 72 h. The synergistic effect for cisplatin is presented in Table I (Q>1.15). The inhibition of the combined group was significantly greater than the single group. The Q value (Q>1.15) implied the two drugs can produce a synergistic effect.

### Detection of cell apoptosis

**Figure 2:**
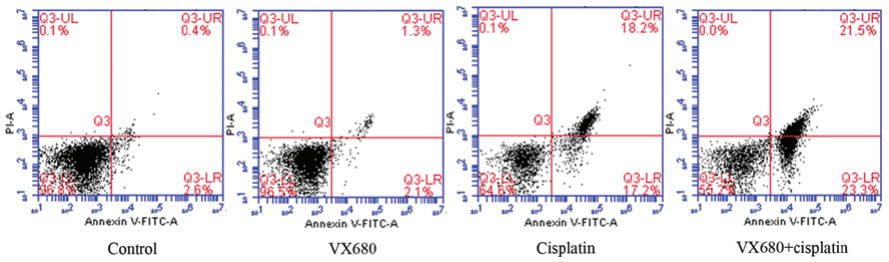
Cell apoptosis in the different groups. Control group, no drugs; single drug group, VX680 (3.125 μmol/l) or cisplatin (0.5 μg/ml); combined group, VX680 (3.125 μmol/l) plus cisplatin (0.5 μg/ml). Cisplatin induced apoptosis in hepatocellular carcinoma (HepG2) cells compared with the control group, but VX680 did not. Cisplatin combined with VX680 significantly increased the apoptosis rate (P<0.05).

HepG2 cell apoptosis was detected using flow cytometry. Compared with the control group, the VX680 group (3.125 μmol/l) presented no significant change in apoptosis rate. However, the apoptosis rate in the combined group was significantly higher than that in the cisplatin group (0.5 μg/ml) and control group (P<0.05; Fig. 2).

### Effect of VX680 and cisplatin on Aurora A, p53 and Bcl-2 protein expression

**Figure 3:**
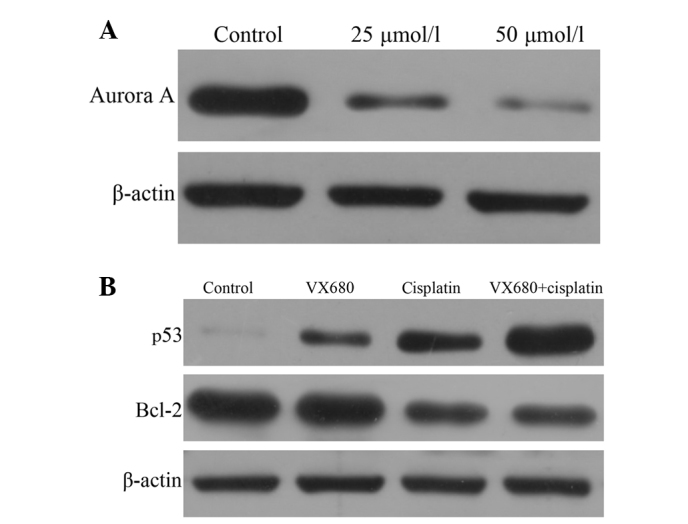
(A) Hepatocellular carcinoma (HepG2) cells were cultured with varying concentrations of VX680 (25 and 50 μmol/l) for 24 h. All cells were collected and analyzed by western blot analysis with an anti-Aurora A antibody. (B) HepG2 cells were treated with 3.125 μmol/l VX680, 0.5 μg/ml cisplatin or a combination of the two for 72 h. Cell lysates were collected and analyzed by western blot analysis with anti-p53, anti-Bcl-2 and anti-β-actin antibodies. The protein level for each group was compared with that of the control group.

VX680 significantly reduced Aurora A expression in a concentration-dependent manner (Fig. 3A). Compared with the control group, cisplatin reduced Bcl-2 expression and increased the expression level of p53 protein (P<0.05). However, VX680 only increased the expression of p53 (P<0.05) and did not reduce the expression of Bcl-2. Bcl-2 and p53 expression levels were significantly reduced and increased, respectively, in the combined group compared with the single drug and control groups (P<0.05; Fig. 3B).

## Discussion

Several studies have indicated that Aurora kinase is overexpressed in the majority of hepatocellular carcinoma tissue samples and cell lines (16,17). A previous study used VE-465, an analog of VX-680, which significantly reduced Aurora A expression and induced apoptosis in HepG2 cells (18). These findings indicated that Aurora A may serve as a molecular target against HCC. Although the antitumor effect of Aurora kinase inhibitors has been demonstrated, it is unclear whether they effectively enhance the effect of cisplatin chemotherapy on HepG2 cells.

In the present study, we used VX680 to inhibit the expression of Aurora A in HepG2 cells and analyzed the cellular changes using an MTT assay. We found that cisplatin and VX680 inhibited the growth of HepG2 cells. Additionally, the combination of VX680 and cisplatin had a synergistic effect (Q>1.15). This result suggests that the suppression of Aurora A expression enhances the sensitivity to cisplatin. Cell apoptosis detection revealed that VX680 alone (at a low concentration) does not induce apoptosis of tumor cells, but cisplatin alone does. When cisplatin was combined with VX680, the apoptosis rate of HepG2 cells increased significantly. Numerous studies have indicated that inhibiting Aurora kinase expression may increase the chemosensitivity of cancer cells (11,12). The present study was consistent with these previous studies.

Moreover, western blotting results revealed that chemosensitivity was associated with the expression of p53 and Bcl-2 proteins. In the control group, the expression of p53 protein was at a low level; however, when VX680 or cisplatin were added, the p53 expression increased. The expression of p53 markedly increased in the combined group.

The p53 gene inhibits the growth of tumor cells by inducing cell cycle arrest or apoptosis, and also increases the chemosensitivity of hepatocellular carcinoma (19). Furthermore, Aurora A is a key regulatory component in the p53 pathway. Overexpression of Aurora A leads to degradation of p53 (20). Thus, VX680 increases the expression of p53 and increases the chemosensitivity of HepG2 cells by increasing the expression of Aurora A. Cell apoptosis was associated with the expression of Bcl-2. The anti-apoptosis activity was reduced, while the chemosensitivity to cisplatin was enhanced.

In conclusion, our results indicate that VX680 inhibits the growth of HepG2 cells and enhances the chemosensitivity of HepG2 cells to cisplatin. Thus, the selective inhibition of Aurora A by VX680 provides a new approach to anticancer therapy and may serve as a single or combined agent with existing therapies in the future.

## Figures and Tables

**Table I:** Inhibitory effect of VX680 combined with cisplatin on HepG2 cells.

VX680 (μmol/l)	Cisplatin (μg/ml)	Inhibition rate (%)	Q-value
3.125	0	7.87±1.08	
0	0.125	17.29±1.93	
0	0.25	26.75±1.27	
0	0.5	37.19±2.37	
0	1	50.41±4.50	
0	2	67.54±5.68	
3.125	0.125	30.61±1.95	1.29
3.125	0.25	42.86±1.72	1.32
3.125	0.5	57.37±2.35	1.36
3.125	1	70.07±2.12	1.29
3.125	2	81.41±3.10	1.16
